# Involvement of Rab28 in NF-κB Nuclear Transport in Endothelial Cells

**DOI:** 10.1371/journal.pone.0056076

**Published:** 2013-02-14

**Authors:** Jun Jiang, Ying-Xin Qi, Ping Zhang, Wen-Tian Gu, Zhi-Qiang Yan, Bao-Rong Shen, Qing-Ping Yao, Han Kong, Shu Chien, Zong-Lai Jiang

**Affiliations:** 1 Institute of Mechanobiology and Medical Engineering, School of Life Sciences and Biotechnology, Shanghai Jiao Tong University, Shanghai, China; 2 Institute of Engineering in Medicine, and Departments of Bioengineering and Medicine, University of California San Diego, La Jolla, California, United States of America; King’s College London, University of London, United Kingdom

## Abstract

Our previous proteomic analysis revealed the expression of Rab28 in arteries of rats. However, the function of Rab28 in mammalian cells, and its role in vessels are still unknown. Coarctation of abdominal aorta above left kidney artery in rat was used as hypertensive animal model. FX-4000 cyclic strain loading system was used to mimic the mechanical condition on vascular cells during hypertension in vitro. Immunofluorescence and co-immunoprecipitation (Co-IP) were used to identify distribution and interaction of Rab28 and nuclear factor kappa B (NF-κB). Rab28 expression was significantly increased in carotid arteries of hypertensive rats. High cyclic strain induced Rab28 expression of endothelial cells (ECs) through a paracrine control of vascular smooth muscles cells (VSMCs), which at least partly via angiotensin II (Ang II). Rab28 knockdown decreased proliferation of ECs, while increased apoptosis and migration. Immunofluorescence revealed that Ang II stimulated the co-translocation of Rab28 and NF-κB from cytoplasm into nucleus. Knockdown of Rab28 attenuated NF-κB activation. Co-IP of NF-κB p65 and Rab28 indicated their interaction. Our results revealed that Rab28, as a novel regulator of NF-κB nuclear transport, might participate in the disturbance of EC homeostasis.

## Introduction

Vascular endothelial cells (ECs), which form the inner surface of blood vessel wall, serve important homeostatic functions in maintaining the vascular physiological states. EC functional changes, such as abnormal permeability, proliferation, apoptosis, alignment, production of chemotactic molecules, and expression of adhesion molecules, etc., play significant roles in many vascular diseases [1], [2]. ECs are exposed to mechanical stimuli in vivo, including shear stress caused by the dragging frictional force of blood flow, and cyclic strain resulting from the repetitive deformation of the cells as the arterial wall rhythmically distends and relaxes with the pulsatile pressure. It has been shown that physiological mechanical stimuli are essential to EC homeostasis, while pathological mechanical stimuli contribute to the development of vascular disorders during hypertension, atherosclerosis, thrombosis, in-stent restenosis, and bypass graft occlusion, etc. [3].

In the pathological process of hypertension, cyclic mechanical strain subjected to the arterial wall increases accordingly. Cyclic strain of brachial arteries is about 5% in normal state and can be elevated to 15% in hypertension [4], [5]. Abundant evidence reveals that abnormal growth and survival of ECs play key roles in vascular remodeling during hypertension [6], [7], and elevated cyclic strain exerts complicated effects in this process [8]–[10].

To evaluate the mechanism involved in EC functional changes during hypertension, we focus on a novel molecule with potential mechano-sensitivity, Rab28, which was firstly revealed by our previous vascular proteomic study [11]. By using coarctation of abdominal aorta hypertensive animal model, we found that the expression of Rab28 was significant increased in the common carotid arteries of hypertensive rats, in comparison to the sham controls (Figure S1). It is reported that Rab28 assists the activity of retromer-dependent lysosome trafficking and ESCRT-mediated lysosome degradative pathways in trypanosomes [12], but its function in mammalian cells is still unknown [13]–[16]. Hence, we hypothesized that Rab28 might be a novel regulator of EC homeostasis and play a significant role in cyclic strain-induced vascular remodeling during hypertension.

Rab family is the largest family of small Ras-like GTPase with more than 60 members in human [17], [18]. It has been reported that most of the Rab GTPases transfer between inactive/active states by their GDP/GTP cycling [19], and act as molecular “switches” for the formation, transport, tethering, and fusion of vesicles, and regulating their traffic between organelles [20], [21]. However, the locations, membrane traffic pathways, functions, and relation to diseases of Rab28 remain unknown.

To evaluate the role of increased Rab28 expression in vessels during hypertension, the cyclic strain loading system was used to mimic the mechanical situation of hypertension in vitro, and to evaluate the role of cyclic strain-modulated Rab28 expression on EC functions. This study provided novel information on the expression, intracellular distribution, and functions of Rab28 in ECs. Understanding of the mechanobiological mechanisms of Rab28 on EC homeostasis will help to define the molecular mechanisms underlying vascular remodeling.

## Results

### Rab28 Expression in Cultured VSMCs and ECs Under Cyclic Strain in vitro

**Figure 1:**
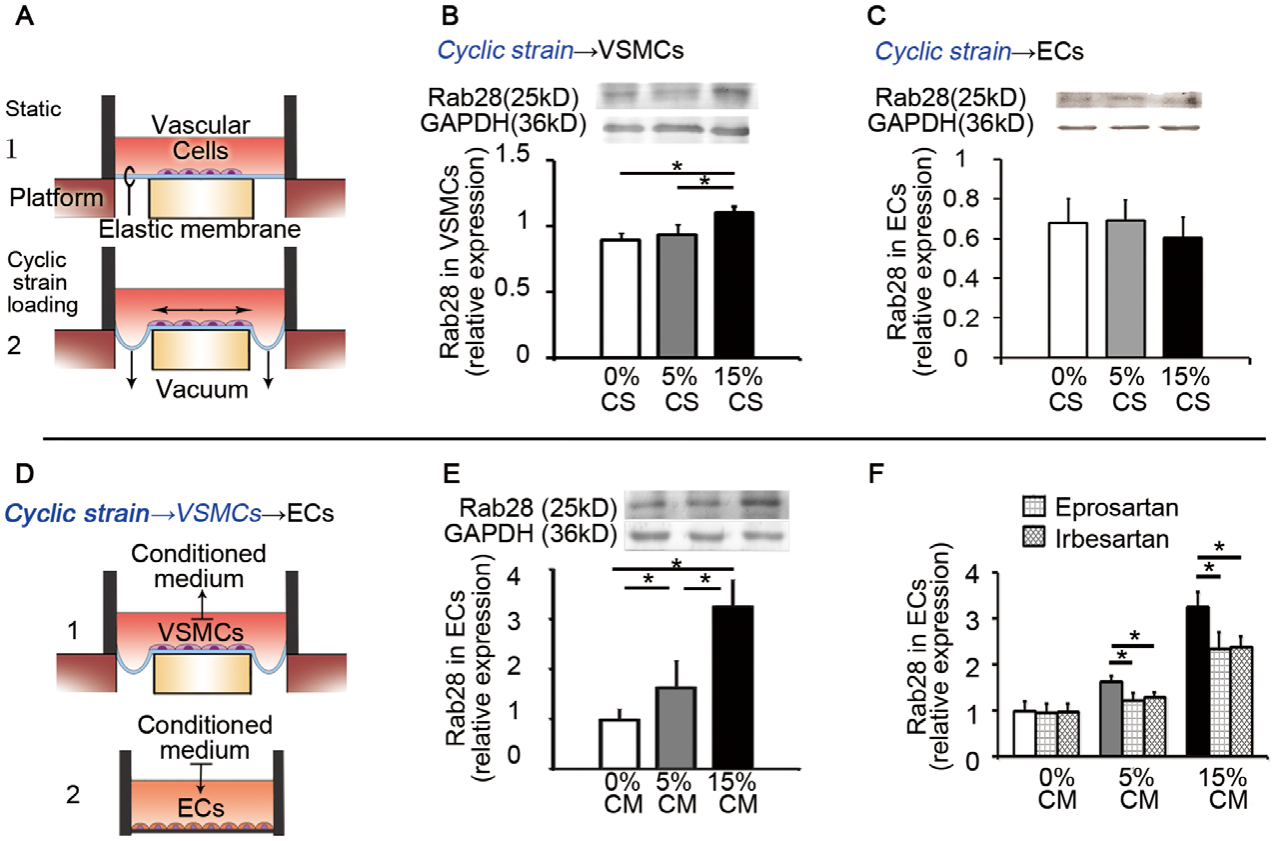
Cyclic strain regulated Rab28 expression in vascular cells in vitro and the conditioned media (CM) from VSMCs induced Rab28 expression in ECs. (**A**) Schematic drawing of the cyclic strain loading system in vitro, FX-4000. Vascular cells (VSMCs or ECs) were seeded on the elastic membrane of the culture plate. A pump produced periodic vacuum beneath the membrane to create the cyclic strain with elongation of the vascular cells. (**B**) Compared with the static group (0% elongation), Rab28 expression in VSMCs was not significantly altered by 5% cyclic strain, but increased significantly by 15% cyclic strain. (**C**) In ECs, the expression of Rab28 was not directly changed by cyclic strain application. (**D**) Schematic drawing of the CM experiments. The CM from VSMCs under different cyclic strain applications was transferred to static cultured ECs. (**E**) The Rab28 expression in ECs was significantly increased in response to the CM from VSMCs subjected to 15% cyclic strain, compared with those from 5% and 0% cyclic strain. (**F**) The induced expression of Rab28 in ECs by CM was attenuated by AT1R blockers, 10^−6^ mol/L Irbesartan and 10^−5^ mol/L Eprosartan. All results are given as mean ± *s.d.*, *P<0.05, *n* = 7 each. CS, cyclic strain; CM, conditioned medium.

VSMCs and ECs cultured from rat aorta were subjected to normal cyclic strain (physiological, 5% elongation at 1.25 Hz) and high cyclic strain (pathological, 15% elongation at 1.25 Hz) for 24 hours, respectively (Figure 1A).

Rab28 expression of VSMCs was very low in both the static (0% elongation) and the physiological 5% cyclic strain group. While the pathological 15% cyclic strain significantly increased the Rab28 expressions of VSMCs in comparison to the static as well as 5% cyclic strain (Figure 1B). In ECs, the expression of Rab28 did not show significant difference among the static, 5% and 15% cyclic strain groups (Figure 1C).

It has been shown that interaction between ECs and VSMCs via paracrine control or direct contact plays a vital role in vascular homeostasis [11], [22], [23]. Hence, we tested the effect of conditioned medium (CM) from VSMCs subjected to cyclic strain on the static ECs, and also the effect of CM from strained ECs on the static VSMCs (Figure 1D). The results revealed that compared with the static control, Rab28 expression in ECs was significantly increased by the CM from VSMCs subjected to cyclic strain, and the increase in 15% strain group was significantly higher than 5% strain (Figure 1E). In contrast, Rab28 expression in VSMCs did not show any significant change after treated with the CM from ECs subjected to 0, 5, or 15% cyclic strain (data not shown).

### Angiotensin II type 1 Receptor (AT1R) Blockers Attenuate the CM-induced Rab28 Expression

Angiotensin II (Ang II) is one of the most important vasoactive molecules secreted from vascular tissue in situ and plays key roles in vascular remodeling through paracrine effects during hypertension [24], [25]. We hypothesized that Ang II might be the crucial molecule that participated in the modulation of Rab28 expression in ECs by the CM from VSMCs subjected to high cyclic strain. ELISA revealed that Ang II concentration in the CM from VSMCs increased in a strain-amplitude dependent manner. The physiological 5% cyclic strain caused an increase in Ang II secretion from VSMCs in comparison to the static control, and the pathological 15% cyclic strain caused a much greater increase in Ang II secretion (data not shown). The expression of Rab28 in ECs subjected to CM from VSMCs was attenuated by both 10^−6^ mol/L Irbesartan and 10^−5^ mol/L Eprosartan, the specific AT1R blockers (Figure 1F).

Rab28 expression in ECs was also induced by exogenous Ang II stimuli in a concentration-dependent manner (Figure S2).

### Role of Rab28 in EC and VSMC Functions

**Figure 2:**
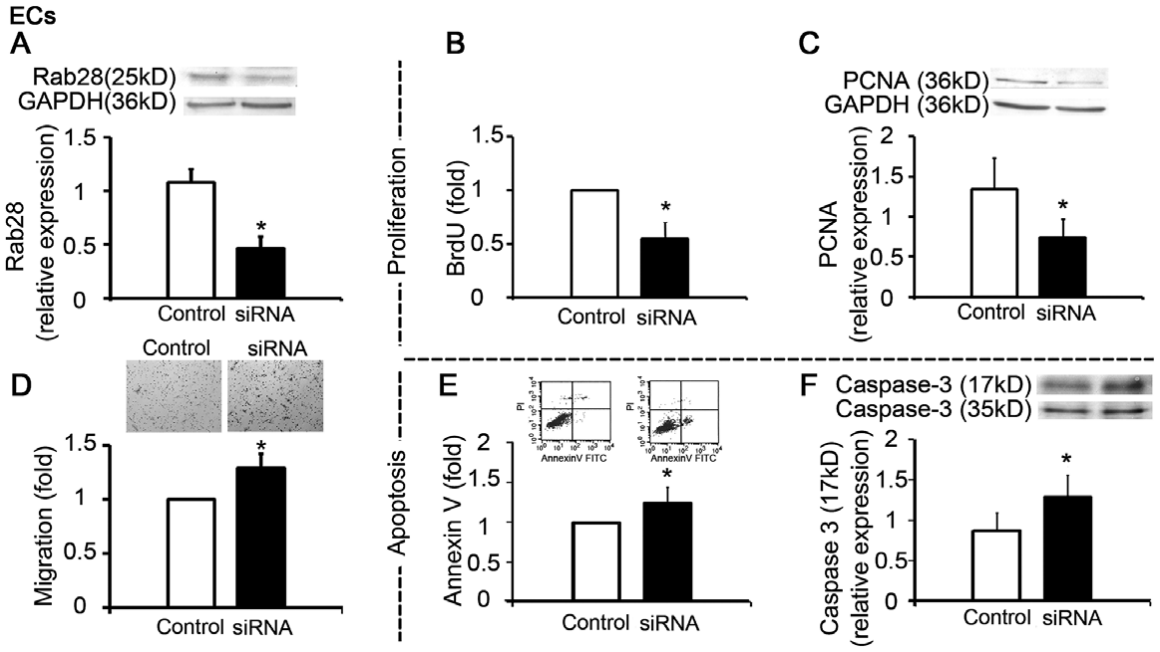
Rab28 knockdown changed cell proliferation, apoptosis and migration in ECs. (**A**) Target siRNA transfection down-regulated the expression of Rab28. (**B, C**) The repressed expression of Rab28 decreased the proliferation, (**D**) increase the migration, (**E, F**) and apoptosis of ECs. All results are given as mean ± *s.d.*, *P<0.05, *n* = 7 each.

**Figure 3:**
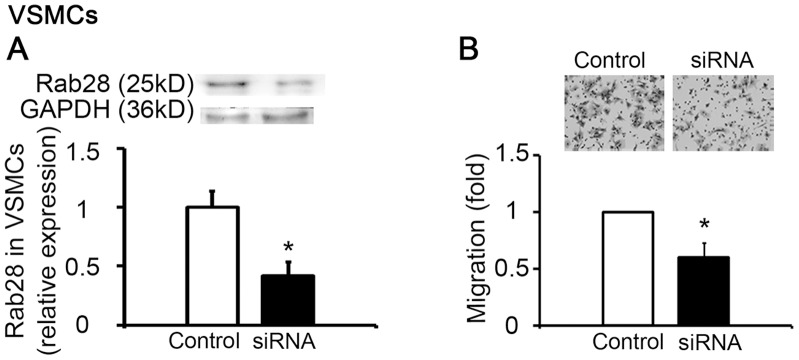
Rab28 knockdown changed cell migration in VSMCs. (**A**) Target siRNA transfection down-regulated the expression of Rab28. (**B**) The repressed expression of Rab28 decreased the migration of VSMCs. All results are given as mean ± *s.d.*, *P<0.05, *n* = 7 each.

To evaluate whether the changed expression of Rab28 participated in the modulation of vascular cell functions, the expression of Rab28 was “knocked-down” by target siRNA transfection, and the migration, proliferation and apoptosis of ECs and VSMCs were then analyzed. The results revealed that the suppressed expression of Rab28 in ECs reduced their proliferation, but enhanced apoptosis and migration (Figure 2A to F). In VSMCs, the suppressed expression of Rab28 impaired migration (Figure 3A, B), but had no specific effect on apoptosis and proliferation (data not shown).

The induced expression of Rab28 in ECs by the CM from VSMCs subjected to 15% cyclic strain was accompanied by a significant increase of EC proliferation and decrease of apoptosis (Figure S3).

### Intracellular Distribution of Rab28 in ECs

Since all well-studied Rab GTPases are localized in the cytoplasm and related to vesicle traffic [20], [21], the dye FM 4-64FX was used to visualize the intracellular vesicles of ECs, and then the cells were fixed and incubated with Rab28 antibody. Immunofluorescence indicated that Rab28 did not co-localize with vesicles in the cytoplasm (Figure S4) and Rab28 existed not only in the cytoplasm, but also in the nucleus of ECs (Figures. S4, S5).

**Figure 4:**
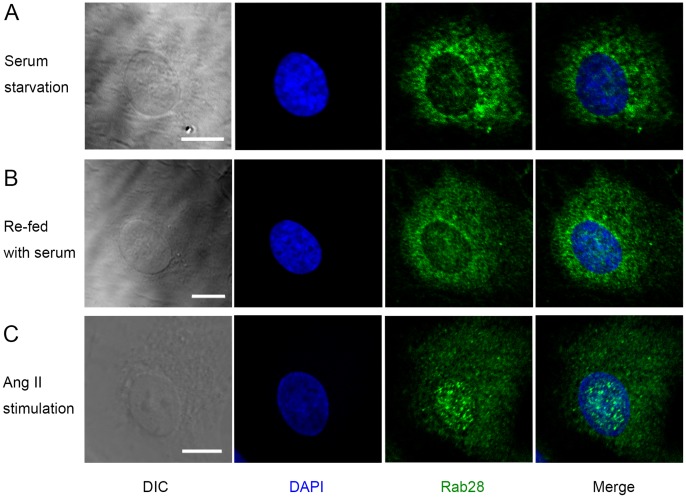
Intracellular distribution of Rab28 in ECs under different culture conditions. (**A**) ECs were starved in serum-free medium for 24 hours to growth arrest. Rab28 existed mainly in the cytoplasm. (**B**) ECs were re-fed with serum for 12 hours. Rab28 appeared in both the cytoplasm and the nucleus. (**C**) ECs were re-fed with serum and treated with 1×10^−6^ mol/L Ang II. Rab28 was distributed mainly in the nucleus. Scale bars: 10 µm.

After synchronizing the ECs with serum-free medium for 24 hours, Rab28 mainly existed in the cytoplasm (Figure 4A). When re-feeding ECs with a medium containing 20% serum, Rab28 appeared in both the cytoplasm and the nucleus again (Figure 4B). Stimulation of ECs with Ang II at a concentration of 10^−6^ mol/L caused a significant translocation of Rab28 from the EC cytoplasm into the nucleus (Figure 4C). Since it has been shown that nuclear factor kappa B (NF-κB) has a similar translocation as Rab28 after Ang II stimuli, and NF-κB is a crucial regulator of EC functions [26], [27], the possible relationship between Rab28 and NF-κB was further examined.

### Co-localization and Functional Correlation of Rab28 with NF-κB in ECs

**Figure 5:**
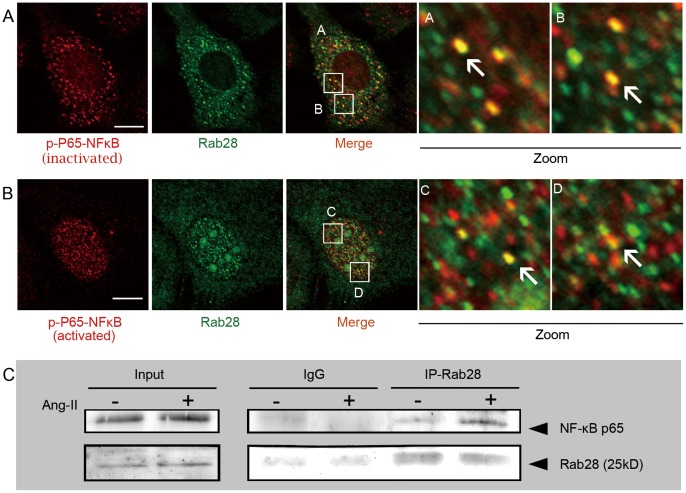
Co-localization and co-immunoprecipitation of NF-κB and Rab28 in ECs. (**A**) NF-κB and Rab28 were co-localized, at least partly, in the cytoplasm of starved ECs. (**B**) Stimulation of ECs with exogenous angiotensin II (Ang II) caused the translocation of both Rab28 and activated NF-κB into the nucleus, with these two molecules still partially co-localized. Scale bars: 10 µm. (**C**) Whole cell lyastes of ECs treated with 10^−6^ M Ang II were prepared and probed for co-immunoprecipitation assays with anti-Rab28 antibody. Proteins from the immunoprecipitateds were detected by Western blot using anti-NF-κB p65 antibody. NF-κB co-immunoprecipitated with Rab28.

Confocal microscopy revealed the co-existence of Rab28 and NF-κB in the cytoplasm in the synchronized ECs (Figure 5A). Ang II stimulated the translocation of both Rab28 and NF-κB from the cytoplasm into the nucleus (Figure 5B). The degree of co-localization of Rab28 and NF-κB in the nucleus and in the cytoplasm is 71±13.5%, which suggested that Rab28 might assist NF-κB nuclear importing. To investigate the interaction between NF-κB and Rab28, ECs were treated with 10^−6^ mol/L Ang II, and the whole cell lysates were prepared and probed with anti-Rab28 antibody. Western blot revealed that NF-κB p65 co-immunoprecipitated with Rab28 (Figure 5C).

**Figure 6:**
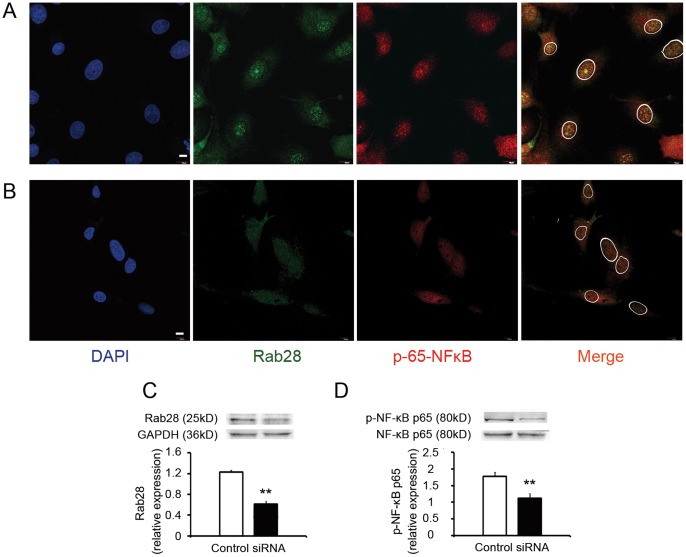
Decrease of Rab28 expression attenuated activation of NF-κB in ECs. (**A**) As a control, ECs were incubated with Lipofectamine 2000 and negative control siRNA. Then cells were stimulated with 10^−6^ mol/L Ang II for 30 minutes. Fluorescence microscopy showed the translocation of NF-κB from cytoplasm into nucleus (activation) was significant. (**B**) ECs were incubated with Lipofectamine 2000 and target siRNA of Rab28. Then cells were stimulated with 10^−6^ mol/L Ang II for 30 minutes. Fluorescence microscopy showed the attenuated Rab28 expression and NF-κB translocation. (**A**) and (**B**) were photographed with the same exposure time. Scale bars: 10 µm. (**C**) siRNA effectively knocked down the expression of Rab28. (**D**) After the knockdown of Rab28, the phosphorylation of NF-κB p65 (activation) were significantly attenuated. All results are given as mean ± *s.d.*, **P<0.01, *n* = 7 each.

In order to assess the interactions between Rab28 and NF-κB, Rab28 expression was knocked down in ECs. Down-regulation of Rab28 by RNA interference attenuated translocation of NF-κB into the nucleus (Figure 6A, B) and the phosphorylation (Figure 6C, D), which suggested that Rab28 participated in the activation of NF-κB.

## Discussion

Long-term hypertension causes vascular remodeling, which is characterized by thickening of vascular media, decreasing of inner-radius-to-thickness ratio, and imbalance of cell proliferation and apoptosis. In the present research, we demonstrated that the pathological cyclic strain up-regulated the expression of Rab28 of ECs via the paracrine effect of VSMCs, which might contribute to the disturbance of EC homeostasis.

Target siRNA transfection significantly decreased Rab28 expression, which down-regulated proliferation and up-regulated apoptosis and migration of ECs, and suppressed migration of VSMCs. The effects of Rab28 on biological behaviors of vascular cells, especially on EC growth and survival, suggest that the modulations of Rab28 expression might participate in vascular remodeling during hypertension.

To prove this hypothesis and evaluate the possible mechanisms by which Rab28 regulates cell functions, the Flexcell cyclic strain loading system was used to simulate the mechanical condition applied to ECs and VSMCs during hypertension [4], [5]. Such an in vitro system provides precise control of the physicochemical environment for the investigation of molecular alternations in the mechanotransduction system, and allows precise measurements of consequent cellular functions. Pathological cyclic strain (15%) directly increased Rab28 expression in VSMCs, but not in ECs. Rab28 expression in ECs, however, increased significantly by the addition of culture CM from VSMCs subjected to the pathological cyclic strain. Combined with the effects of Rab28 siRNA transfection on EC functions, our results indicate that Rab28 expressed in ECs is susceptible to paracrine control by VSMCs and subsequently regulate EC homeostasis. We then investigated what is the bioactive substance in the CM from VSMCs that mediate the paracrine control of ECs.

Ang II is one of the most important bioactive molecules secreted from VSMCs in situ. It has been proven that Ang II plays key roles in vascular remodeling during hypertension [24], [25]. Our present results revealed that Ang II concentration in the CM from VSMCs subjected to pathological cyclic strain is higher than that from VSMCs subjected to physiological cyclic strain. Furthermore, AT1R blockers attenuate the effect of CM from the strained VSMCs on the static ECs. These results indicate that VSMCs subjected to pathological cyclic strain might release Ang II to modulate Rab28 expression in ECs via a paracrine effect, which consequently affect EC functions.

Generally, Rab GTPases act as “traffic switches” between organelles, and their cellular distributions are related to their function. Therefore, we investigated the shuttling of Rab28 between cellular compartments in ECs. Cytoplasm-nucleus shuttling, but not cytoplasm-organelles shuttling, of Rab28 is detected in ECs induced by Ang II. It is known that with Ang II stimulation, NF-κB dissociates from the inhibitor of NF-κB (IκB) to become activated [26] and the activated NF-κB then translocates into the nucleus to bind to chromosome to induce gene transcription [28].

Our results indicate that both Rab28 and NF-κB positively influence EC proliferation, while negatively regulate EC apoptosis and migration. Furthermore, Rab28 might assist the nuclear transport of NF-κB to regulate EC functions, in view of (a) the similar cytoplasm-nucleus translocation of both Rab28 and activated NF-κB, (b) the co-localization of Rab28 and NF-κB in both the cytoplasm and nucleus, (c) co-IP of Rab28 and NF-κB p65, and (d) the roles of the other Rab GTPase family members in intracellular trafficking.

**Figure 7:**
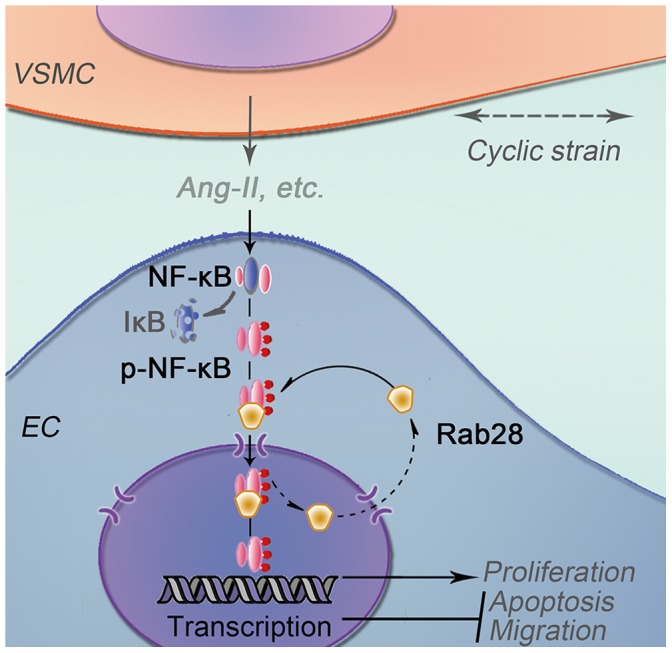
Schematic drawing outlines the possible role of Rab28 in EC homeostasis. Pathological cyclic strain loading on VSMC activates the local angiotensin system. VSMC secretes Ang II into the intercellular space (conditioned medium). Ang II induces the expression and translocation of Rab28 in ECs, which aids the NF-κB nuclear transporting and activation. Then the activated NF-kB eventually modulates gene transcription in charge of EC proliferation, apoptosis and migration.

Ang II and NF-κB play important roles in vascular cell proliferation and apoptosis in the presence of mechanical force. The effects of Ang II on cell proliferation are augmented by mechanical stretch in VSMCs of spontaneously hypertensive rat [29]. Activation of NF-κB in arteries subjected to high intraluminal pressure prevents apoptosis in vascular cells [30]. Our findings demonstrate that the Ang II synthesized by VSMCs subjected to high cyclic strain mediates the increase of Rab28 expression and the NF-κB activation in ECs. Rab28 increases the activation of NF-κB and then translocates with it into the nucleus. The activation of NF-κB eventually modulates gene transcription to modulate EC proliferation, apoptosis and migration (Figure 7).

Large molecules (>40 kD) cannot pass through nuclear envelope freely. Their nuclear transport is controlled by the nuclear pore complex (NPC) [31]–[33]. Proteins need karyopherins, including importins and exportins, to enter or leave the nucleus, respectively [34], [35]. Importins require nuclear localization signals (NLS) to identify which proteins could enter into the nucleus, while exportins search for nuclear export signals (NES) to identify which proteins could leave from the nucleus. As a result, karyopherin-cargo protein complex can pass through NPC. The separation of importin-cargo protein complex or combination of exportin with a cargo protein requires Ran GTPase [36], a nuclear Ras, to change the conformation of importin or exportin. Our present results suggested that, in addition to Ran, Rab28 might also regulate nuclear transport.

Taken together, our results suggest that Rab28 might modulate EC proliferation, apoptosis and migration through its assistance of NF-κB translocation from the cytoplasm into the nucleus. This study has revealed novel information on the expression, intracellular distribution and functions of Rab28. Understanding of the effects of Rab28 on molecular “switches” and biological function will help to define the molecular mechanisms underlying vascular homeostasis and development of vascular pathologies, such as atherosclerosis, thrombosis, hypertension, as well as their clinical complications.

## Materials and Methods

All procedures involving animals conform to the Guide for the Care and Use of Laboratory Animals published by the US National Institutes of Health (NIH publication no. 85–23, revised 1996), and were approved by the Animal Research Committee of Shanghai Jiao Tong University.

### Cell Culture and Cyclic Strain Application

The SD rats were euthanized at the end of the experiments with sodium pentobarbital at 120 mg/kg. After the animal was sacrificed, the thoracic aorta was surgically removed. ECs and VSMCs were isolated from the thoracic aorta of healthy SD rat as previously described [11], [22], [23]. The cells were seeded on the elastic membrane of a culture plate (BioFlex, Oakland Park, FL, USA) at a density of 3×10^5^ per plate (diameter 9.32 cm^2^).

The cells were subjected to cyclic strain provided by a cyclic strain loading system FX-4000 (Flexcell international, Hillsborough, NC, USA), with an elongation magnitude of 5% at a frequency of 1.25 Hz to simulate the physiological vascular environment, and 15% at 1.25 Hz to simulate the hypertensive environment.

ECs in the vessel wall in vivo are adjacent to VSMCs, and there is closely functional interrelation between them. To study the reciprocal paracrine interactions between ECs and VSMCs in the condition of different cyclic strain, the CM of VSMCs subjected to different magnitudes of cyclic strain were transferred to the static ECs, and the CM of ECs subjected to cyclic strains were transferred to the static VSMCs.

### Cell Proliferation and Apoptosis Assay

Vascular cells were seeded on the 96-well plate after being subjected to cyclic strains and those treated with CM. The cells were incubated with bromodeoxyuridine (BrdU) (Basel, Roche, Switzerland) for 8 hours. The proliferation level of vascular cells was detected with a BrdU antibody (Roche) followed by an enzyme-linked immunosorbent assay (ELISA) protocol. Proliferating cell nuclear antigen (PCNA) detection by Western blot (PCNA antibody. Sigma, St. Louis, MO, USA) was also used to determine cell proliferation.

The cells subjected to cyclic strains and those treated with CM were gently digested from the original plates and re-suspended. The cells were then incubated with Annexin V-FITC (R&D systems, Minneapolis, MN, USA) and propidium iodide (PI). Cell apoptosis level was detected by a flow cytometry (FACS Calibur. Becton Dickinson, Franklin Lakes, NJ, USA). Detection of caspase-3 by Western blot (Caspase-3 antibody. Cell Signaling, Danvers, MA, USA) was also used to assess cell apoptosis.

### Detection of Ang II in the CM and Blockade of AT1R

The CM from the VSMCs subjected to cyclic strains was transferred to 96-well plates. Ang II concentrations in the media were detected with an Ang II EIA kit (Phoenix Pharmaceuticals, Burlingame, CA, USA).

To block the effect of Ang II, ECs were pre-treated with AT1R blockers, Irbesartan and Eprosartan, for 60 min. Then the CM from VSMCs was used to incubate ECs.

To study the effects of Ang II on the Rab28 expression in ECs, exogenous Ang II was added to the static ECs.

### Rab28 Knockdown in ECs and VSMCs

To evaluate the role of Rab28 on the functions of vascular cells, the expression of Rab28 was “knocked-down” by target small interfering RNAs (siRNA) transfection. The strands of siRNA for Rab28 were synthesized (GenePharma, Shanghai, China): 5′-GGCAAGAUGUUGGAUAAAUTT-3′, 5′-AUUUAUCCAACAUCUUGCCTT-3′. The siRNA were transfected to ECs with Lipofectamine 2000™ (Invitrogen, Carlsbad, CA, USA).

Rab28 expression was detected by Western blot with anti-Rab28 antibody (Abcam, Cambridge, UK) and the activation level of NF-κB was detected by Western blot with anti-phosphor-NF-κB p65 antibody (Sigma, USA).

Cell migration was detected by using the Transwell system (Costar, USA). Cell proliferation was evaluated by BrdU-ELISA and PCNA expression. Cell apoptosis was evaluated by Annexin V-FITC flow cytometry and caspase-3 expression.

### Intracellular Distribution of Rab28 and its Co-localization with NF-κB in ECs

In order to elucidate the mechanism by which Rab28 regulates proliferation, apoptosis and migration of ECs, the intracellular distribution and translocation of Rab28 was determined. ECs were cultured under serum-deprivation, normal growth condition, and Ang II stimulation, respectively. Rab28 protein distributions in ECs under different conditions were studied by immunofluorescence (anti-Rab28 antibodies, Abcam) using a confocal microscope (FV1000. Olympus, Shinjuku, Tokyo, Japan).

Based on the distribution of Rab28 in ECs, Rab28 and NF-κB were double-stained with their respective antibodies. Ang II, 10^−6^ mol/L, was used to stimulate ECs for 12 hours.

### Co-immunoprecipitation (Co-IP) of NF-κB p65 and Rab28

ECs were incubated with 10^−6 ^mol/L Ang II for 1 hour and lysed with cold RIPA lysate on ice. After centrifugation, anti-Rab28 antibody was added to the corresponding supernatants and incubated overnight at 4°C. Then Protein G plus/Protein A agarose suspension (Calbiochem, Switzerland) was added to the samples and incubated 2 hours at 4°C. Then the products were separated by 12% SDS-PAGE and NF-κB p65 was detected with its antibody (Cell signaling, Danvers, MA,USA).

### Statistical Analysis

Data are expressed as mean ± *s.d.* Results between groups were compared by one-way ANOVA and Fisher’s least significant difference (LSD) test. Differences were considered statistically significant when the *P*-value was <0.05.

See Text S1 for the detailed materials and methods.

## Supporting Information

Figure S1 **Rab28 expression was elevated in the common carotid arteries from the hypertensive rats.**
(TIF)

Figure S2 **Exogenous Ang II up-regulated the expression of Rab28 in ECs.**
(TIF)

Figure S3 **The conditioned media (CM) from VSMCs induced EC proliferation and protected them from apoptosis.**
(TIF)

Figure S4 **Intracellular vesicles and Rab28 were double-labeled in ECs.**
(TIF)

Figure S5 **Rab28 distributed in the nucleus of ECs.**
(TIF)

Text S1 **The detailed materials and methods.**
(DOC)
