# Supplementary figures and images for: Involvement of Rab28 in NF-κB Nuclear Transport in Endothelial Cells
Source: PLoS One. 2013 Feb 14;8(2):e56076. doi: 10.1371/journal.pone.0056076 (PMC3573041; doi:10.1371/journal.pone.0056076)

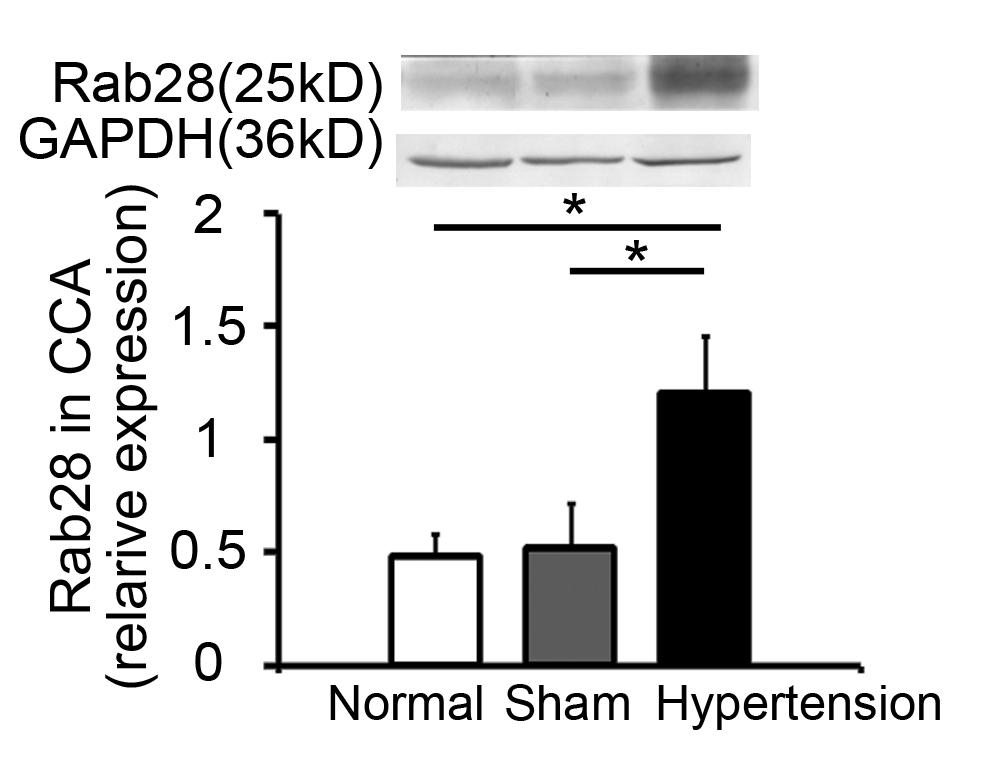

Supplement: Figure S1 — Rab28 expression was elevated in the common carotid arteries from the hypertensive rats. (TIF) [file pone.0056076.s001.tif]

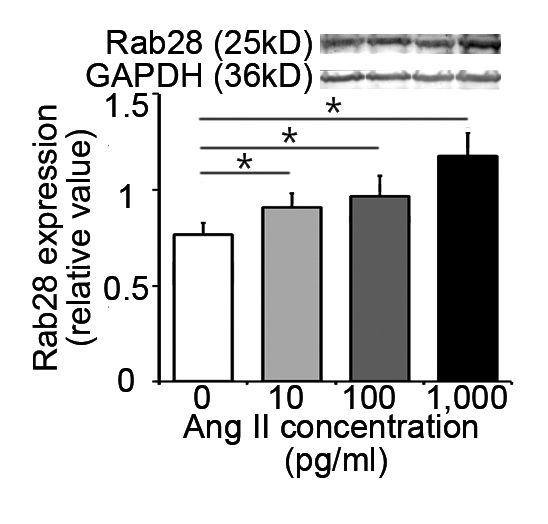

Supplement: Figure S2 — Exogenous Ang II up-regulated the expression of Rab28 in ECs. (TIF) [file pone.0056076.s002.tif]

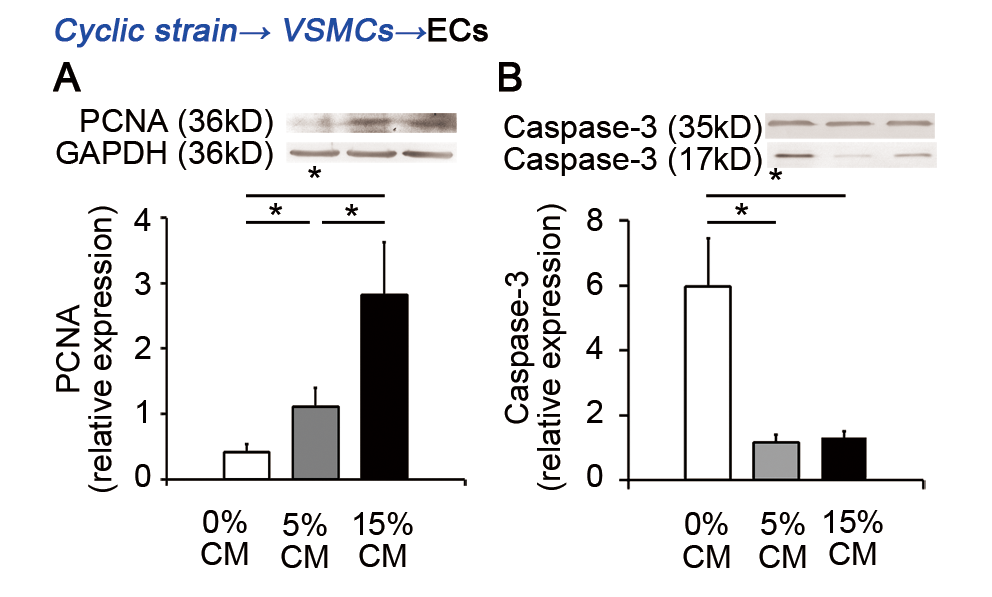

Supplement: Figure S3 — The conditioned media (CM) from VSMCs induced EC proliferation and protected them from apoptosis. (TIF) [file pone.0056076.s003.tif]

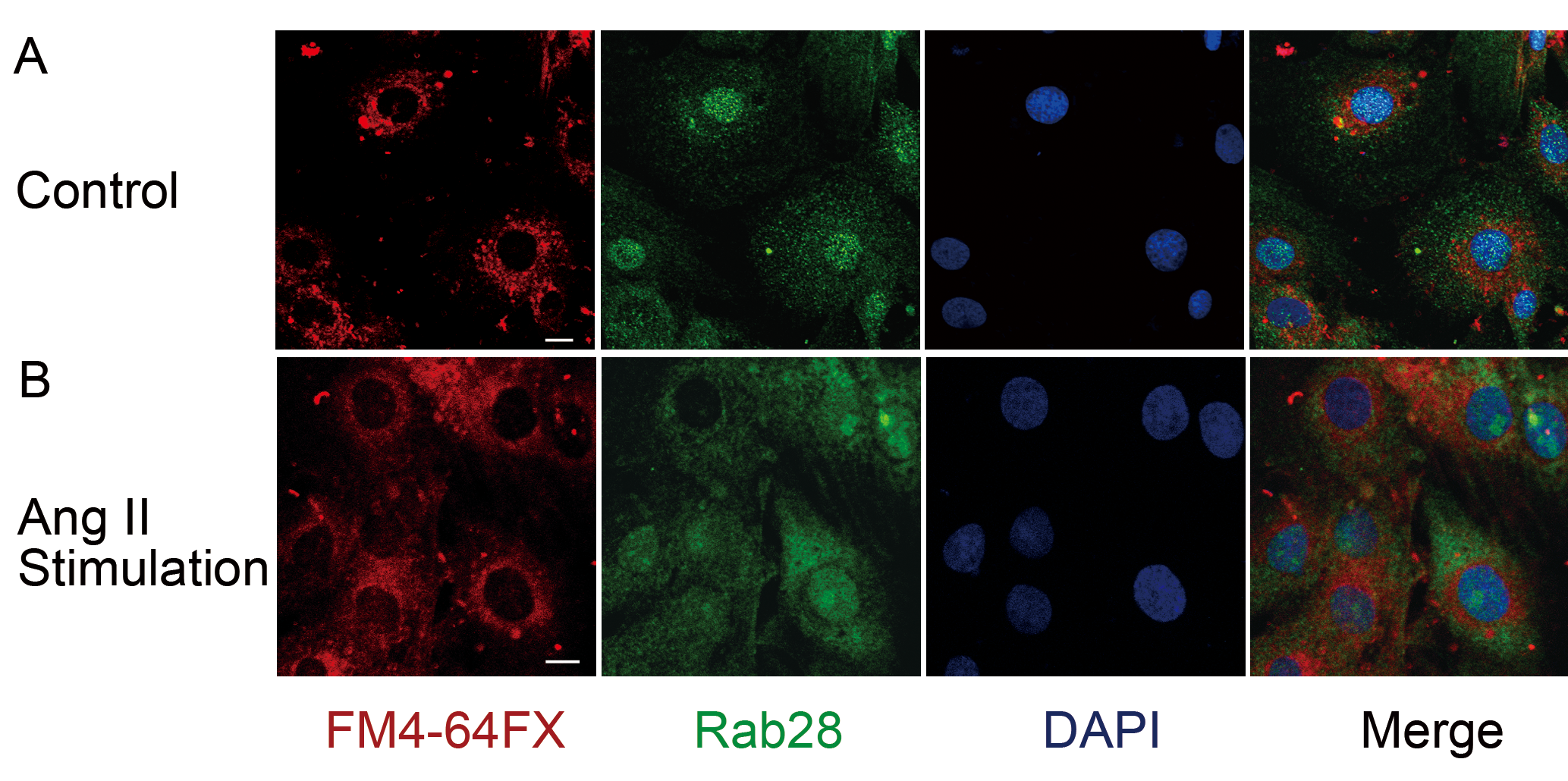

Supplement: Figure S4 — Intracellular vesicles and Rab28 were double-labeled in ECs. (TIF) [file pone.0056076.s004.tif]

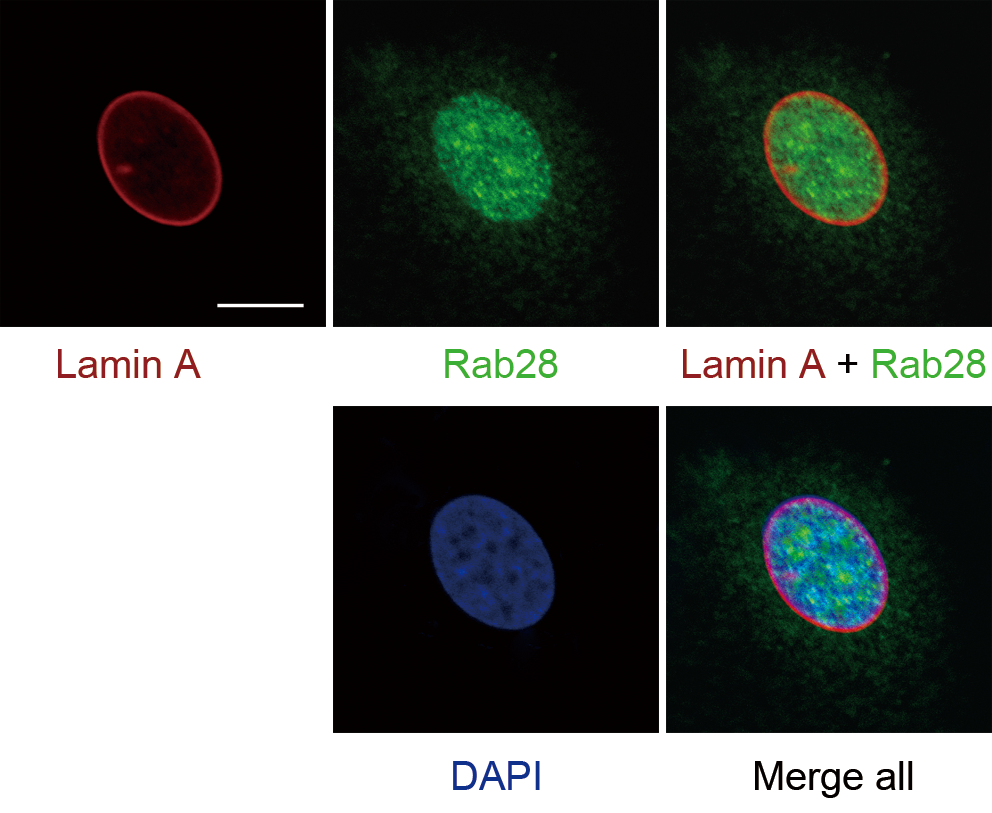

Supplement: Figure S5 — Rab28 distributed in the nucleus of ECs. (TIF) [file pone.0056076.s005.tif]
