# Supplementary material for: Involvement of Rab28 in NF-κB Nuclear Transport in Endothelial Cells
Source: PLoS One. 2013 Feb 14;8(2):e56076. doi: 10.1371/journal.pone.0056076 (PMC3573041; doi:10.1371/journal.pone.0056076)
Supplement: Text S1 — The detailed materials and methods. (DOC) [file pone.0056076.s006.doc]

PONE-D-12-34442R1 10.1371 / journal.pone.0056076

**Jiang, et al. Involvement of Rab28 in NF-κB nuclear transport in endothelial cells**

**Supporting Information**

**Text S1. The detailed materials and methods**

**Hypertension model**

Eight-week-old male Sprague-Dawley (SD) rats weighing 200 ± 15 g were randomly assigned to un-operated, sham-operated and abdominal aorta constriction groups. Animals of sham-operated and abdominal aorta constriction groups were anaesthetized with an intraperitoneal injection of pentobarbital sodium (40 mg/kg). The adequacy of anesthesia was evaluated by monitoring the respiration, heart rate and hind limb reflexes. In the abdominal aorta constriction group, the abdominal aorta was dissected at 5 mm above the branch of left kidney artery, and then a 21-gauge needle (outer diameter 0.82 mm) was placed beside the aorta. Thenceforth, a 3-0 (diameter 0.2 mm) suture was tightly bound around the aorta and the needle. The needle was then removed, thus leading to severe aortic constriction . In the sham-operated group, the animal was treated as same as the abdominal aorta constriction group except the aortic constriction. Blood pressure of all animals was measured after 8 weeks. The rats were anaesthetized with sodium pentobarbital at 40 mg/kg and measured blood pressure with a common carotid artery intubation. All the abdominal aorta-coarctated rats exhibited hypertension with systolic pressure > 160 mmHg (21.28 kPa). The un-operated and sham-operated rats were normotensive with systolic pressure below 120 mmHg (15.96 kPa). The rats were euthanized with sodium pentobarbital at 120 mg/kg then the full-length of common carotid arteries were dissected, isolated, and frozen sectioned to determine the morphological parameters. The artery specimen was ground with liquid nitrogen and total protein extraction was obtained by the lysis buffer. Rab28 protein expression in the full-thickness-tissue of commonly carotid arteries was detected by Western blot.

After 8 weeks of abdominal aorta coarctation, the operated rats retained a stable level of hypertension, whereas the blood pressure in the sham controls was not significantly different from the un-operated control. The common carotid arteries in the hypertensive rats demonstrated signs of vascular remodeling, including thickening of vascular media, decrease of inner-radius-to-thickness ratio, and increase of cell proliferation in vascular media (data not shown).

**Cell culture and cyclic strain loading**

The healthy male SD rats weighing 200 ± 15 g were euthanized with sodium pentobarbital at 120 mg/kg. After the animal was sacrificed, the thoracic aorta was surgically removed. Primary cultured ECs were isolated from the thoracic aorta intima with 0.2% type I collagenase and were seeded in Medium 199 (M199, Gibico) containing 20% fetal bovine serum (FBS, Gibico) . ECs were characterized by immunocytochemical staining with Von-Willebrand factor (vWF) antibody (Sigma). VSMCs were isolated from sheared tunica media of the thoracic aorta and were seeded in Dulbecco’s modified Eagle’s medium (DMEM, Gibico) containing 10% FBS . VSMCs were identified by immunocytochemical staining with smooth muscle-specific α-actin (SMA) antibody (Sigma).

ECs with 2-4 passages and VSMCs with 4-7 passages, respectively, were seeded on type I collagen-coated elastic membrane of BioFlex culture plate (Flexcell international, US) at a seeding density of 3×10^5^ per plate (diameter 9.32 cm^2^) . When the cells fully spread on the plate, 2 mL serum-free M199 for ECs and 2 mL serum-free DMEM for VSMCs were added to synchronize cells for 12 hours. The cells were then subjected to cyclic strain provided by computer-controlled vacuum of the Flexcell cyclic strain loading system (FX-4000 Strain Unit, Flexcell international, US). The cyclic strains were set to an elongation magnitude of 5% at a frequency of 1.25 Hz to simulate the physiological vascular environment and an elongation of 15% at 1.25 Hz to stimulate the hypertensive environment, for 24 hours. After mechanical loading, the EC conditioned medium (CM) were transferred to the static cultured VSMCs, and the VSMC CM were transferred to the static cultured ECs, respectively.

**Cell proliferation and apoptosis assay**

To detect proliferation, cells were seeded on 96-well plate at the density of 1×10^4^ per well and Bromodeoxyuridine (BrdU, Roche) was added to the culture medium to a final concentration of 1 μmol/L. After 8 hours incubation at 37°C in a humidified atmosphere (5% CO_2_), the cells were fixed and labeled with BrdU antibody (Roche). After the addition of colorimetric substrate (POD), the absorbance of the samples was measured with an ELISA reader (BioRad). Proliferating cell nuclear antigen (PCNA) detection by Western blot also was used to indicate cell proliferation (PCNA antibody, Sigma).

To determine apoptosis, cells were gently digested from original plates and re-suspended, and then collected by centrifugation and washed in cold PBS. Annexin V-FITC (Trevigen, R&D) and propidium iodide (PI) were used to incubate cells in the dark for 15 minutes at 25°C. Samples were processed by flow cytometry (FACS Calibur, BD). Caspase-3 detection by Western blot also was used to indicate cell apoptosis (Caspase-3 antibody, Cell Signaling).

**Detection of angiotensin II (Ang II) in the CM and blockade of Ang II type 1 receptor (AT1R)**

ECs were seeded on 6-well plates to spread out fully on the plates. Nutrient culture medium (with 20% FBS) was replaced with serum-free medium for 24 hours for synchronization. 0 pg/ml, 10 pg/mL (10^-8^ mol/L), 100 pg/mL (10^-7^ mol/L), 1,000 pg/mL (10^-6^ mol/L) and 10,000 pg/mL (10^-5^ mol/L) of Ang II (Sigma) were used to stimulate ECs for 12 hours. 1,000 pg/mL (10^-6^ mol/L) was used in the subsequent experiments. To block AT1R of ECs, ECs were pre-treated with 10^-6^ mol/L Irbesartan and 10^-5^ mol/L Eprosartan for 60 min respectively. Then the CM form VSMCs was used to incubate ECs.

**Cell migration assay**

Transwell system (Costar) was used to detected cell migration. The polycarbonate membrane of Transwell contains 8-μm diameter pores, which allow cells to migrate through. VSMCs were suspended in serum-free DMEM and ECs were suspended in serum-free M199 at a final concentration of 1.0 × 10^6^ cells/mL, before seeding onto the upper-side of the membrane. The medium of the underside of the membrane contained 20% FBS as source of chemotaxis. After 6 hours for VSMCs and 12 hours for ECs, the cell layer above the membrane was removed with a cotton swab. The cell layer below the membrane was fixed and stained, and then countered under an inverted phase contrast microscope (Olympus).

**Intracellular distribution of Rab28 and its co-localization with NF-κB in ECs**

For the co-localization analysis, ECs were seeded at 50 - 60% confluence to spread out on the coverslips. Serum-free M199 was used to starve the cells for 24 hours. ECs kept in the serum-free medium for another 12 hours, re-fed with 20% FBS for 12 hours, and stimulated with 10^-6^ mol/L Ang II in 20% FBS M199, respectively. All groups were fixed in 4% formaldehyde and permeated with 0.5% Triton X-100, then incubated with mouse anti-Rab28 polyclonal antibody (1:200) in 5% bovine serum albumin (BSA).

To determine the spatial relation between Rab28 and NF-κB, mouse anti-Rab28 (1:200) and rabbit anti-phosphor-NF-κB p65 (ser536) monoclonal antibodies (1:200, Cell Signaling) were used to double stain Rab28 and NF-κB.

**Co-immunoprecipitation (Co-IP) of NF-κB p65 and Rab28**

ECs (1×10^6^) were challenged with 10^-6^ mol/L Ang II for 1 hour and lysed with 500 μl RIPA lysate (1% NP-40, 0.25% deoxycholate, Byotime) and 1 mmol/L PMSF (Byotime) on ice. The cell lysates were centrifuge at 12,000 rpm for 30 minutes at 4°C and remove the supernatants to the new tubes. 1 μg mouse IgG or 1 μg anti-Rab28 antibody was added to the corresponding supernatants and incubated overnight at 4°C. Then 30 μl Protein G plus/Protein A agarose suspension (Calbiochem) was added to the samples and incubated for 2 hours at 4°C. Finally, IP and co-IP products were rinsed with cold RIPA and further lysed with 0.1% SDS and 1% TritonX-100 lysate, separated by 12% SDS-PAGE. NF-κB p65 was detected with its antibody (Cell signaling).

**Rab28 knockdown**

To knock down Rab28 expression in vascular cells, the strands of small interfering RNAs (siRNA) for Rab28 (NCBI reference sequence NM-053978) were synthesized. The siRNA (100 pmol) were transfected to 1×10^6^ ECs with Lipofectamine 2000^TM^. After incubating the cells at 37°C, 5% CO_2_ for 24 hours, cells were lysed with 0.1% SDS and 1% TritonX-100 lysis buffer. Finally the cell lysate was separated by 12% SDS-PAGE and the expression of Rab28 was detected by Western blot with its antibody. Nonsilencing siRNA that does not recognize any known homology to rat genes was used as negative control.

**Statistical analysis**

Immunofluorescence images were measured by the Image-Pro Plus software. Results of Western blot were quantitative analyzed by using BioRad Quantity One. All values are expressed as mean ± *s.d*.. Results between groups were compared by one-way ANOVA and Fisher’s least significant difference (LSD) test. A value of *P* < 0.05 was considered statistically significant.

**References**

1. Barton CH, Ni Z, Vaziri ND (2001) Enhanced nitric oxide inactivation in aortic coarctation-induced hypertension. Kidney Int 60: 1083-1087.
2. Kwan HY, Leung PC, Huang Y, Yao X (2003) Depletion of intracellular Ca2+ stores sensitizes the flow-induced Ca2+ influx in rat endothelial cells. Circ Res 92: 286-292.
3. Redmond EM, Cullen JP, Cahill PA, Sitzmann JV, Stefansson S, et al. (2001) Endothelial cells inhibit flow-induced smooth muscle cell migration: role of plasminogen activator inhibitor-1. Circulation 103: 597-603.
4. Liu B, Qu MJ, Qin KR, Li H, Li ZK, et al. (2008) Role of cyclic strain frequency in regulating the alignment of vascular smooth muscle cells in vitro. Biophysical Journal 94: 1497-1507.
5. Sharifpoor S, Simmons CA, Labow RS, Paul Santerre J (2011) Functional characterization of human coronary artery smooth muscle cells under cyclic mechanical strain in a degradable polyurethane scaffold. Biomaterials 32: 4816-4829.
